# Correlation between systemic branched-chain amino acid metabolism and tear fluid inflammation with visual quality of life in patients with dry eye disease

**DOI:** 10.3389/fimmu.2026.1817802

**Published:** 2026-10-16

**Authors:** Yingming Wang, Lijuan Que, Zhengtai Sun, Lei Zhong, Xiaofeng Zhang

**Affiliations:** 1 Department of Ophthalmology, The First Affiliated Hospital of Soochow University, Suzhou, Jiangsu, China; 2 Department of Ophthalmology, The Fourth Affiliated Hospital of Soochow University, Suzhou, Jiangsu, China

**Keywords:** branched-chain amino acids, clinical signs, correlation analysis, disease severity, dry eye disease, tear inflammatory factor, untargeted metabolomics

## Abstract

**Background:**

Dry eye disease (DED) is a common multifactorial ocular surface disorder characterized by tear film instability, inflammation, and visual impairment, with rising global prevalence affecting quality of life. While inflammation is central to DED pathophysiology, the role of systemic metabolic dysregulation in modulating local ocular surface inflammation remains unclear. This study investigates the correlation between systemic branched-chain amino acid (BCAA) metabolism and tear inflammatory markers as well as visual-related quality of life (VR-QOL) in DED patients.

**Methods:**

This retrospective study enrolled 111 DED patients and 64 healthy controls from a single center (2022–2024). In the pilot phase, tear samples from 26 DED and 22 controls underwent untargeted metabolomics using UPLC-MS to identify differential metabolites and pathways. In the serum-based sequential exploratory phase, serum BCAA levels (leucine, valine, ILE) were measured in 85 DED patients (stratified by severity) and 42 controls. Tear inflammatory factors (IL-1β, IL-6, IL-8, hs-CRP, TNF-α) were quantified by ELISA/immunofluorescence. Pearson correlation analyzed associations between serum BCAAs and ocular surface sign parameters (Schirmer’s test, corneal staining), and inflammatory markers. Receiver operating characteristic (ROC) curve analysis was performed to evaluate the apparent discriminative ability of serum BCAAs for suboptimal visual quality of life in patients with dry eye disease (DED).

**Results:**

Untargeted metabolomics revealed 15 significantly altered tear metabolites in DED (8 upregulated, 7 downregulated). Enrichment analysis revealed a suggestive trend toward enrichment of the BCAA biosynthesis pathway (Raw P = 0.016; after Benjamini–Hochberg correction, FDR Q = 0.058, which did not meet the significance threshold of Q < 0.05.). In this study, pathways with raw P < 0.05 but corrected Q ≥ 0.05 were defined as exhibiting a “suggestive enrichment trend” and were not considered as definitive conclusions, warranting further targeted validation. Serum levels of leucine, valine, and ILE were significantly elevated in both mild-to-moderate and severe DED groups versus controls (all P<0.05), but did not differ by severity. Positive correlations were found between each BCAA and specific tear inflammatory factors (IL-1β, hs-CRP, TNF-α; r=0.35–0.66, all P<0.05), while no significant associations emerged with IL-6, IL-8, or clinical signs. ROC analysis revealed notable associations between serum BCAAs and poor VR-QOL, with AUCs of 0.916 (leucine), 0.837 (valine), and 0.886 (ILE). These exploratory findings suggest a potential link between BCAA levels and vision-related quality of life, but do not establish clinical utility as biomarkers.

**Conclusions:**

Untargeted tear metabolomics in a small cohort revealed BCAA-related metabolic alterations in DED. Sequential exploratory analysis of an independent large-sample serum cohort further demonstrated that elevated serum Leu, Val, and ILE, which correlated positively with tear IL-1β, hs-CRP, and TNF-α at the population level. These findings provide exploratory evidence linking serum BCAAs to DED. However, given the use of two distinct matrices without paired sampling and the control group sample size n = 42 during the serum association exploratory phase being modestly lower than the predetermined goal—potentially compromising the statistical power of some tests—the results only support population-level cross-biofluid associations and do not confirm individual-level coupling.

## Introduction

Dry eye disease (DED), a multifactorial ocular surface disorder characterized by the loss of tear film homeostasis as its core feature, commonly presents with symptoms including ocular dryness, foreign body sensation, asthenopia, and visual fluctuation in affected patients (1–3). The incidence of this condition has risen markedly in recent years, with substantial adverse effects on patients’ quality of life and visual function (4, 5). The pathogenesis of DED involves multiple mechanisms, including ocular surface inflammation, aberrant neural regulation, increased tear osmolarity, and alterations in sex hormone levels; however, the role of systemic metabolic factors in this process remains incompletely understood (6). Therefore, exploring systemic metabolic correlates of DED is of great significance for advancing the etiological understanding of this condition and expanding early intervention strategies.

Amino acids serve as essential substrates for protein synthesis and participate in both physiological and pathological processes. Branched-chain amino acids (BCAAs), in particular, exert a variety of biological functions, including promoting protein synthesis, regulating blood glucose, and alleviating fatigue. Mechanistically, BCAAs competitively inhibit protein hydrolases, thereby reducing circulating levels of free essential amino acids and subsequently stimulating protein synthesis. Moreover, BCAAs have been shown to up-regulate glucose transporter expression, enhance insulin secretion, and facilitate glucose uptake into cells for oxidative metabolism. (7, 8). Furthermore, branched-chain amino acids (BCAAs) exert both excitatory and inhibitory effects on the central nervous system. By competing with aromatic amino acids—such as phenylalanine and tryptophan—for transport across the blood–brain barrier, BCAAs modulate neurotransmitter levels in the brain, thereby influencing cognitive function and behavior (9).

In recent years, accumulating evidence has demonstrated that dysregulated branched-chain amino acid (BCAA) metabolism is implicated in the pathogenesis of various diseases. Decreased BCAA levels have been associated with liver cirrhosis, urea cycle disorders, and chronic kidney disease, whereas elevated BCAA concentrations are closely linked to the development of obesity and both type 1 and type 2 diabetes mellitus (10). In addition, it has been reported that inappropriate use of branched-chain amino acid supplements may induce neuronal excitotoxicity and oxidative stress, which may pose potential health risks (11). Epidemiological studies have indicated diabetes as one of the important risk factors for the development of DED, which may be attributed to ocular surface cellular edema, corneal neuropathy, and lacrimal gland dysfunction under hyperglycemic conditions (12, 13). This suggests a potential biological link between systemic metabolic disturbances and DED. Therefore, some researchers suggest that monitoring systemic BCAAs metabolism may serve as a novel diagnostic biomarker for DED. Consistent with this notion, several clinical studies have reported alterations in the tear metabolomic profiles of DED patients in recent years (14–16). However, most studies have focused on local tear metabolite differences, with limited exploration of the relationship between systemic amino acid metabolism and ocular surface inflammation, and insufficient rigorous cross-sample validation (e.g., tears and serum) of specific candidate metabolites. The generalizability of untargeted tear metabolomics findings requires further validation. In this study, we attempted to integrate untargeted metabolomic analysis of tear fluid with targeted detection of serum BCAAs, employing a progressive exploratory design with two independent cohort designs to perform a progressive cross-biofluid correlation analysis between tear metabolic profiles and serum BCAA levels in DED patients, with the aim of exploring the associations of systemic BCAA metabolism with ocular surface inflammation and vision-related quality of life.

## Materials and methods

### Study design and participant flow

This was a single-center retrospective observational study involving 175 participants recruited from the First Affiliated Hospital of Soochow University between June 2022 and December 2024. The study comprised two phases: the pilot phase included 48 participants (26 DED patients and 22 healthy controls undergoing ophthalmic examinations). This phase was designed to conduct a preliminary exploratory analysis of tear metabolic profiles in DED patients using a small sample size, with the aim of screening for potential aberrant metabolic pathways. The serum-based sequential exploratory phase included 127 participants (85 DED patients and 42 healthy controls). This phase was designed to expand the sample size and to perform a cross-matrix association analysis of the previously identified BCAA-related metabolic alterations in an independent serum cohort, thereby assessing the robustness of these metabolic changes at the systemic level. This study was a retrospective observational study, and the research protocol was approved by The First Affiliated Hospital of Soochow University (Approval No. 2024-146), and all procedures were performed in accordance with the ethical standards of the 1964 Declaration of Helsinki and its later amendments. This study involved a retrospective analysis of pre-existing clinical data and biospecimens (serum and tear samples collected between June 2022 and December 2024) from 175 patients. No additional invasive procedures or prospective interventions were performed. All data were anonymized prior to analysis, and the study did not alter patients’ diagnostic, therapeutic, or follow-up regimens. Given the retrospective nature of the study and the use of de-identified existing data and biospecimens, the requirement for informed consent was waived by the Ethics Committee. All data were used exclusively for the purposes of this research, with no commercial use or third-party sharing, in compliance with the Personal Information Protection Law and relevant data security regulations.

The analytical subjects in the pilot phase and serum-based sequential exploratory phases of this study did not overlap, and the two phases were based on two distinct biological matrices—tear fluid (untargeted metabolomics) and serum (targeted BCAA quantification), respectively. The relationship between the two stages is best characterized as a progressive “discovery–association exploration” framework, rather than a conventional “discovery–independent validation” paradigm. Given that paired tear–serum BCAA measurements were not performed in the same individuals, this design precludes the establishment of a direct quantitative association at the individual level between systemic BCAA levels and local tear BCAA metabolism. Nevertheless, the two-stage results provide independent evidence derived from distinct biofluids and non-overlapping patient cohorts, suggesting that BCAA metabolic abnormalities may concurrently manifest in both tear fluid and serum, albeit insufficient to confirm a metabolic coupling between systemic and local compartments.

This study comprised two independent phases (**Figure 1**): a pilot phase (n = 48) for tear untargeted metabolomic profiling to screen for differential metabolites and aberrant pathways, and a serum-based sequential exploratory phase (n = 127) for targeted serum metabolomics of BCAAs and subsequent clinical association analyses. The subjects in the two phases did not overlap. As shown in **Figure 1**, both the DED and healthy control groups were well balanced with respect to baseline characteristics, including age and sex, with no significant differences between groups (P > 0.05). In the figure, CON denotes the healthy control group, and DED denotes the dry eye disease group. Numbers in boxes indicate subgroup sample sizes, and arrows indicate the direction of the study workflow. Dashed boxes represent the sample sources and types (tear fluid vs. serum) for each phase, with numbers indicating subgroup sample sizes (n).

**Figure 1:**
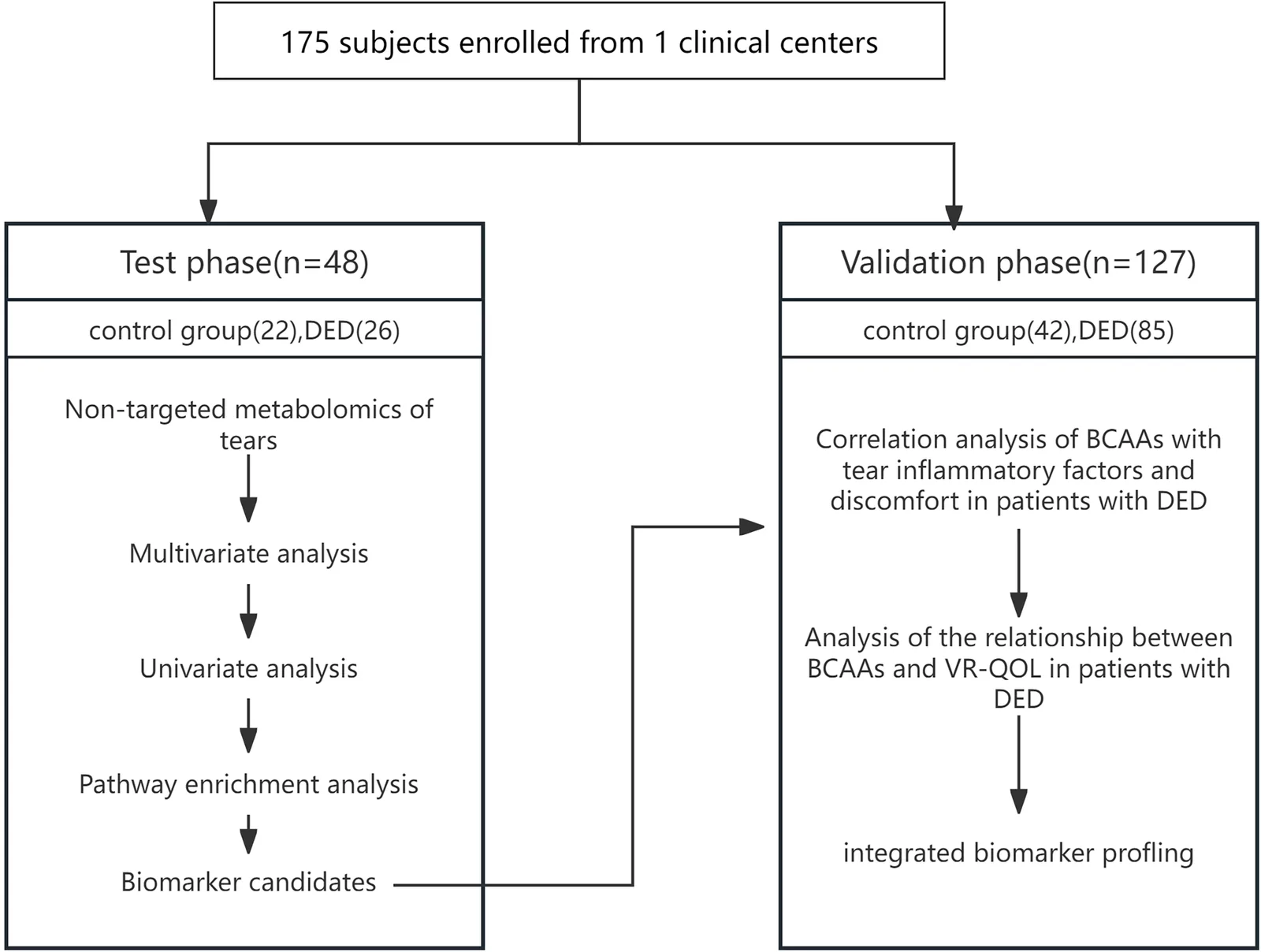
Flow chart of this study.

Sample size determination: This study was an exploratory untargeted metabolomics investigation, for which no well-established method is currently available for precise sample size estimation based on metabolomic effect sizes. For the pilot phase, the sample size (22 controls and 26 DED patients) was designed with reference to comparable tear metabolomics studies, with a minimum of 20 cases per group considered adequate for screening differential metabolites in untargeted metabolomic analyses. For the serum-based sequential exploratory phase (42 controls and 85 DED patients), the sample size was determined based on the following considerations: with a two-sided significance level of α = 0.05 and a statistical power of 1 − β = 0.90, a minimum of 44 subjects per group was required to detect a moderate effect size difference (Cohen’s d ≥ 0.50) in serum BCAAs between the two groups. Ultimately, the serum-based sequential exploratory phase included 85 DED patients and 42 controls, yielding a total of 127 subjects. The sample size for the serum association exploratory phase (42 controls and 85 DED patients) was initially designed to detect a moderate effect size difference (Cohen’s d ≥ 0.50) with α = 0.05 and statistical power (1 − β) = 0.90, which yielded a required minimum of 44 participants per group. However, due to the constraints inherent in the retrospective study design and the availability of the biospecimen repository, the actual number of controls enrolled in this phase was 42, falling marginally short of the prespecified target of 44 per group. Although the DED group (n = 85) sufficiently met this requirement, the slight under-sampling in the control group may have compromised the statistical power of certain tests, particularly for those indicators with effect sizes approaching the lower bound of the moderate range, thereby potentially increasing the risk of false-negative findings. Therefore, when interpreting negative results, the potential impact of inadequate statistical power should be carefully considered. Conversely, for statistically significant positive findings, the robustness of the conclusions is relatively reliable, given that the effect sizes were large and the statistical power was presumably adequate.

### Inclusion criteria and exclusion criteria

Inclusion criteria: (1) Patients in the DED group met the diagnostic criteria as outlined in the *Chinese expert consensus on dry eye: lifestyle-related dry eye (2022) (*17) and were confirmed through specialized ophthalmological examinations; (2) Ages ≥18 years; (3) Absence of concomitant dysfunction of major organs such as the heart, liver, or kidneys; (4) Absence of contraindications to the relevant examinations.

Exclusion criteria: (1) Patients with other ocular diseases that may affect examination results (e.g., keratitis, conjunctivitis); (2) Individuals with a history of ocular trauma or surgery; (3) Patients with severe neurological disorders or cognitive impairment; (4) Patients with comorbid malignant tumors or active systemic infections; (5) Pregnant or lactating women.

Classification of DED severity: In the serum-based sequential exploratory phase, patients with DED were stratified according to the TFOS DEWS II dry eye severity grading system. The DEWS II classification defines four levels of severity: Grade 1 (mild, with symptoms but no signs); Grade 2 (moderate, with mild discomfort and possible minimal staining); Grade 3 (severe, with frequent or persistent symptoms accompanied by obvious ocular surface staining); and Grade 4 (very severe, with disabling symptoms and severe ocular surface damage). For the purposes of this study, Grades 1 and 2 were categorized as the mild-to-moderate group, and Grades 3 and 4 as the severe group. Severity grading was determined independently by two ophthalmologists based on a comprehensive assessment of clinical symptoms, slit-lamp examination, corneal fluorescein staining, and tear film breakup time. Any disagreement was resolved through discussion to reach a consensus.

### Baseline characteristics

This study adopted a retrospective design, with all patient data extracted from the medical record system. The collected variables included: gender, age, body mass index (BMI), disease duration (verified by clinicians through structured interviews), history of underlying diseases, smoking history, and alcohol consumption history. Underlying diseases were defined as follows: diabetes was diagnosed according to the *2019 ESC Guidelines on diabetes, pre-diabetes, and cardiovascular diseases developed in collaboration with the EASD (*18), and hypertension was diagnosed based on the *2017 ACC/AHA hypertension guidelines* (19). It should be noted that, due to the constraints of retrospective data collection from medical records, information on several variables—including dietary structure, renal function indicators, medication exposure, topical ophthalmic treatments, contact lens use history, history of autoimmune diseases, and behavioral factors such as screen time—was not available in this study. These variables may independently influence serum BCAA levels and the severity of DED; however, they were not included in the data collection or analysis of the present study.

### Untargeted tear metabolomics analysis

Tear samples (5 μL) were collected from all participants in the small-sample cohort using the capillary collection method. Specifically, sterile, non-irritating glass capillaries with a diameter of 0.3-0.5 mm and a length of 75 mm were gently placed at the tear meniscus of the lower eyelid to draw tears via capillary action. Each participant underwent multiple collections to accumulate 5 μL. After collection, tears were gently expelled from the capillary into pre-chilled cryogenic tubes and immediately stored at -80 °C. Data processing and feature extraction: Raw mass spectrometry data were processed using Thermo Scientific Compound Discoverer 3.3 software for peak extraction, peak alignment, and basic quality control. The peak detection parameters were set as follows: signal-to-noise ratio threshold ≥ 3, mass tolerance ≤ 5 ppm, retention time window of 0.1 min, and minimum peak intensity of 1000 counts. Isotopic peaks and adduct peaks were excluded using the built-in models of the software. After peak alignment, the data matrix was subjected to missing value imputation using the k-nearest neighbors method (k = 5) via the metaX package, followed by unit variance scaling and quantile normalization to eliminate batch effects and enhance data reliability. Quality control (QC) samples (n = 6) were run in each batch, and metabolic features with a peak area RSD > 30% in QC samples were removed. A total of 148 metabolites were finally identified. Blank control samples were used to exclude background ion interference, and features with signal intensity in solvent blanks exceeding 50% of that in the sample were eliminated.

Metabolite identification and confidence level: Metabolite identification was performed in accordance with the confidence level criteria of the Metabolomics Standards Initiative (MSI). MSI Level 1 (confirmed metabolites) was assigned when identification was verified by co-elution with authentic standards (retention time deviation ≤ 0.1 min, mass-to-charge ratio deviation ≤ 5 ppm) and confirmed by MS/MS spectral matching. MSI Level 2 (putatively annotated compounds) was assigned based on matching of accurate mass-to-charge ratio, retention time, and MS/MS fragmentation spectra against the mzCloud public database and an in-house standard spectral library (match score ≥ 80%). All annotated metabolites in this study were identified with confidence levels of no less than Level 2, with Level 1 accounting for approximately 15%. All differential metabolites were further confirmed by MS/MS fragmentation. Pathway enrichment analysis was conducted using the KEGG database, with the significance of enrichment for each pathway assessed by hypergeometric test. The resulting P-values were adjusted using the Benjamini–Hochberg method to control the false discovery rate, with a Q-value < 0.05 considered the threshold for significant enrichment. Pathways with a nominal P-value < 0.05 that did not survive FDR correction were also reported as suggestive findings. Tear metabolomics was performed following a standardized protocol. Tear samples were centrifuged, and the supernatant was analyzed using a UPLC-MS system equipped with a Waters ACQUITY UPLC HSS T3 Column (100 mm × 2.1 mm, 1.8 μm, USA). The chromatographic conditions were as follows: column temperature: 40 °C; mobile phase: 0.1% formic acid in water (A) and 0.1% formic acid in acetonitrile (B). Gradient elution program: 0–2 min (5% B) → 4 min (30% B) → 8 min (50% B) → 10 min (80% B) → 14 min (100% B). Mass spectrometry: Thermo Scientific Q-Exactive HF-X mass spectrometer with electrospray ionization (ESI) source in positive and negative ion modes; scan range: 100–1200 m/z; resolution: 70,000. For MS/MS, resolution was adjusted to 17,500. Key reagents: Methanol and formic acid (chromatographic grade, Thermo Fisher); L-2-chlorophenylalanine (Shanghai Hengchuang) as internal standard.

Technical challenges and variability control in tear sampling: Tear samples were collected using the glass capillary tube method, with approximately 0.5–1 μL collected per single draw, and multiple draws were pooled to obtain 5 μL per participant. This method presents specific technical challenges in patients with DED, as reduced basal tear secretion may prolong the collection time and potentially induce reflex tearing, thereby compromising the representativeness of the sample composition. To minimize sampling variability, the following measures were implemented in this study: (1) all collections were performed by two trained senior technicians at the same time of day to minimize circadian rhythm-related effects; (2) the capillary tube was gently placed at the inferior tear meniscus without touching the cornea or conjunctival surface, so as to minimize reflex secretion; (3) the collection time was controlled within 5 min. Previous studies have demonstrated that tear samples collected via the capillary method exhibit satisfactory intra-day reproducibility in metabolomic analyses. In the present study, all tear samples were processed using an identical collection and handling protocol to minimize operator-related systematic variation.

Metabolite identification was performed in accordance with the confidence level criteria of the Metabolomics Standards Initiative (MSI). Putative annotations were first assigned by matching accurate mass-to-charge ratio (mass tolerance < 5 ppm), chromatographic retention time, and MS/MS fragmentation spectra against the mzCloud public database and an in-house standard spectral library. Among these, metabolites that were further verified by co-elution with authentic standards were assigned a confidence level of MSI Level 1, whereas those identified solely by matching accurate mass, retention time, and MS/MS spectra against the databases were assigned MSI Level 2. All annotated metabolites in this study achieved confidence levels of no less than Level 2, thereby ensuring the reliability and reproducibility of metabolite annotation. Quality control: To ensure the reliability and reproducibility of the metabolomics data, a multi-tiered quality control system was established in this study. (1) Blank controls: blank samples (extraction solvent without biological matrix) were run in each batch to monitor background signals and cross-contamination. (2) Internal standard calibration: an equal amount of L-2-chlorophenylalanine was added to all samples prior to extraction as an internal standard to monitor extraction efficiency and instrument response fluctuations. (3) Quality control (QC) samples: QC samples were prepared by pooling equal aliquots of all sample extracts, and were injected after every 10 sample runs. The base peak intensity and retention time coefficient of variation (CV) of QC samples were used to evaluate system stability. In this study, the QC samples exhibited a retention time CV of < 2% and a peak area CV of < 15% (n = 6), and the QC samples showed a tight cluster in the PCA score plot (blue dots in **Figure 2**), indicating favorable system stability. Metabolic features with a CV ≥ 30% in QC samples were excluded from further analysis.

**Figure 2:**
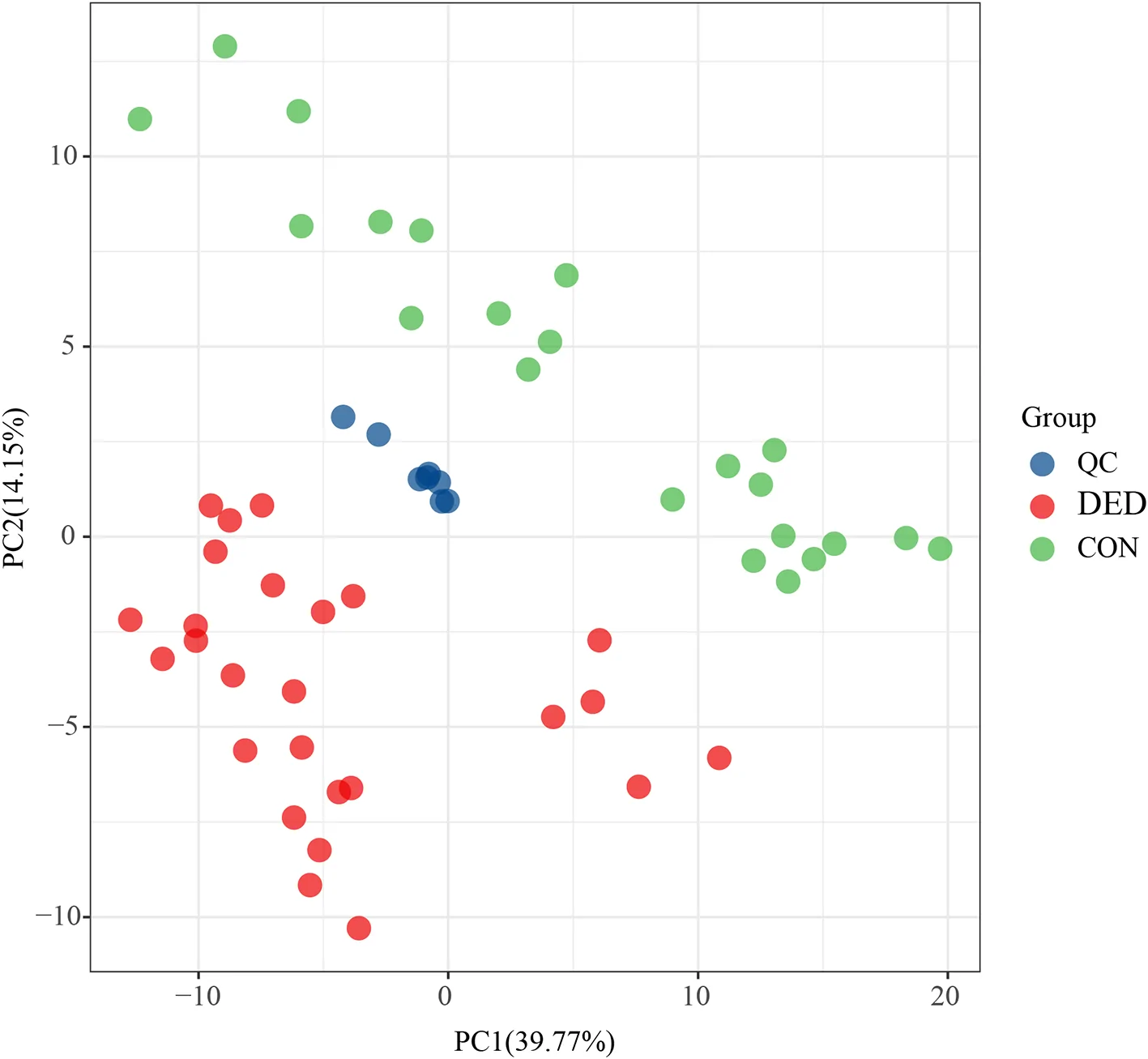
PCA plot of tear sample metabolomics (PCA score plot of tear metabolic profiles in the control and DED groups based on UPLC-MS untargeted metabolomics data (**Figure 2**). The PCA score plot shows the overall separation trend of tear metabolic profiles between the control and DED groups. Principal components PC1 and PC2 explained 28.5% and 16.2% of the total variance, respectively, with ellipses representing the 95% confidence intervals. Green dots: healthy controls (CON, n = 22); red dots: DED patients (n = 26); blue dots: quality control (QC) samples (n = 6), which were prepared by pooling equal aliquots of all sample extracts and injected after every 10 sample runs. The QC samples clustered tightly, indicating good system stability, while the DED samples showed a scattered distribution, suggesting considerable metabolic heterogeneity among patients with DED.).

### Ocular symptom assessment

Schirmer’s test (SIT): All participants in the large-sample cohort were seated upright and instructed to look forward. The physician used tear detection filter strips (Tianjin Jingming New Technology Development Co., Ltd.) by folding one end of the strip, gently pulling down the lower eyelid of the participant, and inserting the folded portion into the middle-third of the lower conjunctival sac. The remaining part of the strip was allowed to hang naturally on the eyelid surface. After 5 minutes, the strip was removed, and the wetting length was measured and recorded.

Corneal fluorescein staining (FL): The physician placed a sodium fluorescein strip in the lower fornix of the conjunctiva. Using a slit lamp, the corneal fluorescein staining was observed. Participants strictly followed instructions to open or close their eyes during the procedure. The cornea was divided into four quadrants centered on the pupil. Staining in each quadrant was scored as follows: 0 points: No staining; 1 point: 0–30 punctate stains; 2 points: >30 punctate stains without fusion; 3 points: Confluent staining. Scores from all quadrants were summed to obtain the total corneal fluorescein staining score for each participant.

### Tear inflammatory factors detection

Tear samples (5 μL) were collected from all participants in the large-sample cohort using the capillary collection method (sterile glass capillaries were gently used to draw tears from the lower conjunctival sac, avoiding ocular irritation to prevent reflex tearing and ensure collection of basal tears). Each participant underwent multiple collections to accumulate 5 μL. Due to the small volume and high concentration of endogenous substances in tear samples, prior to enzyme-linked immunosorbent assay (ELISA) and immunofluorescence testing, the samples were diluted at a 1:10 ratio according to kit instructions. All final concentration values were corrected based on the dilution factor.

Instruments and Reagents: Immunofluorescence measurements were performed using an FS-113 immunofluorescence detector (Wondfo Biotech, China). Tear levels of IL-1β, IL-6, IL-8, and TNF-α were measured using commercially available ELISA kits (IL-1β and IL-8: Beckman Coulter, USA; IL-6: R&D Systems, USA; TNF-α: Chemstan Biological Technology, Wuhan, China) according to the manufacturers’ protocols. Hypersensitive C-reactive protein (hs-CRP) concentrations were determined by immunofluorescence using the dedicated detector. All tear samples were tested within 4 hours of collection.

### Laboratory testing

Fasting peripheral venous blood samples (4 mL) were collected from all participants and sent for testing within 1 hour. After centrifugation at 3,000 rpm for 10 minutes, serum was separated and stored at −80 °C. All laboratory tests were performed according to clinical guidelines (20), and all blood samples were processed within 4 hours of collection.

Complete Blood Count: An automated hematology analyzer (BC5300; Mindray, Shenzhen, China) was used, and the measured parameters included red blood cell count, neutrophil count, and hemoglobin level.

Blood glucose: Fasting blood glucose (FBG) levels were measured using the glucose oxidase method, and glycated hemoglobin (HbA1c) levels were quantified via high-performance liquid chromatography (HPLC). Both assays were performed using the Roche Cobas 8000 system.

Serum BCAAs Detection: Serum levels of leucine (Leu), valine (Val), and ILE (Ile) were quantified using an amino acid automatic analyzer (L-8800; Hitachi, Japan) with manufacturer-provided reagents.

### Statistical treatment

In this study, data were analyzed using IBM SPSS 27.0 (IBM Corp., Armonk, N.Y., USA). Categorical data were expressed as [n] and were compared using the chi-square (x^2^) test. For continuous variables, the Shapiro-Wilk test was applied to assess normality. Non-normally distributed data were expressed as median and interquartile range [M (P_25_, P_75_)] and were compared using non-parametric tests. Normally distributed data were presented as mean ± standard deviation and were compared using the *t* test. A p-value < 0.05 was considered statistically significant. The Pearson correlation coefficient was used to analyze the relationships between BCAAs and both ocular surface clinical parameters and tear inflammatory factors in DED patients. Linear associations were assessed using linear regression. GraphPad Prism 8.3 was utilized for graphical and supplementary statistical analyses. The significance level was set at α = 0.05. For multiple testing correction, the Benjamini–Hochberg method was applied to adjust raw P-values to FDR Q-values in the screening of differential metabolites, with Q < 0.05 set as the threshold for statistical significance. For pathway enrichment analysis, the hypergeometric test was employed, and the resulting raw P-values were corrected to FDR Q-values using the same method, with Q < 0.05 considered the threshold for significant enrichment. Meanwhile, pathways with raw P < 0.05 but Q ≥ 0.05 were objectively reported as suggestive findings and interpreted with caution in the Discussion section. To control for potential confounding, multivariable linear regression models were constructed to evaluate the associations of serum BCAAs (Leu, Val, and Ile) with tear inflammatory cytokines (IL-1β, hs-CRP, and TNF-α), with adjustment for age, sex, BMI, diabetes, and hypertension. For the multivariable logistic regression analysis of VR-QOL, poor VR-QOL was coded as 1 and good VR-QOL as 0; therefore, an OR > 1 indicated increased odds of poor VR-QOL. For differential metabolite screening, raw P-values were adjusted to FDR Q-values using the Benjamini–Hochberg method. Pathway enrichment was assessed using the hypergeometric test against KEGG pathways, with raw P-values adjusted to FDR Q-values via the Benjamini–Hochberg method; a Q-value < 0.05 was adopted as the threshold for significant enrichment, and both the raw P-values and the adjusted Q-values were reported.

Missing data handling: This study was a retrospective analysis, and the overall proportion of missing variables was relatively low. For continuous variables, the missing rate was < 3% (BMI missing in 2 cases, FBG missing in 1 case, HbA1c missing in 1 case), and no missing data were present for categorical variables. For the few missing values in continuous variables, multiple imputation using the MICE algorithm (number of imputations = 5) was performed, with the imputation model including age, sex, BMI, DED severity group, and the primary outcome measures (SIT, FL, and inflammatory cytokines) as auxiliary variables. Importantly, among the 85 patients with DED included in the multivariable linear regression analyses shown in **Table 1**, no missing values were present for any variables entering these models, including serum BCAAs, tear inflammatory cytokines, age, sex, BMI, diabetes history, and hypertension history. Therefore, the **Table 1** regression models were fitted directly to the original complete data from these 85 patients, and Rubin’s-rule pooling across the multiply imputed datasets was not required for the coefficients or standard errors reported in **Table 1**. The adjusted R² and overall F statistics were obtained for the same complete-data model specification. Missing values in the metabolomics data, primarily arising from low-abundance metabolites falling below the limit of detection, were handled using the k-nearest neighbors imputation method (k = 5), after confirming that the proportion of missing values was < 20% and that the missingness was random.

**Table 1:** Multivariable linear regression analysis of the associations between serum BCAAs and tear inflammatory cytokines (n=85).

Dependent variables	Independent variables	Unstandardized B	Standard error	Standardized coefficient	t-value	Significance	95%CI	Adjusted R2	F-value
IL-1β	Leu	0.278	0.076	0.411	3.657	<0.001	0.129~0.427	0.550	18.111
IL-1β	Val	0.539	0.156	0.416	3.461	<0.001	0.233~0.845	0.484	14.132
IL-1β	ILE	0.200	0.059	0.389	3.420	<0.001	0.084~0.316	0.539	17.369
hs-CRP	Leu	2.546	0.705	0.387	3.613	<0.001	1.164~3.928	0.548	17.973
hs-CRP	Val	4.218	1.445	0.335	2.919	0.005	1.386~7.050	0.462	13.022
hs-CRP	ILE	1.998	0.543	0.399	3.677	<0.001	0.934~3.062	0.541	17.501
TNF-α	Leu	0.005	0.020	0.019	0.224	0.823	-0.034~0.044	0.006	1.085
TNF-α	Val	0.009	0.041	0.019	0.207	0.837	-0.071~0.089	0.006	1.085
TNF-α	ILE	0.004	0.016	0.024	0.273	0.785	-0.027~0.035	0.009	1.127

All models were based on 85 subjects in the DED group during the serum association exploratory phase, with adjustment for age, sex, BMI, history of diabetes, and history of hypertension. No missing values were present for any variables included in these nine regression models; therefore, the models were fitted directly to the original complete data from the 85 patients with DED, and no Rubin’s-rule pooling was required. The reported regression coefficients, standard errors, t statistics, P values, confidence intervals, adjusted R² values, and overall F statistics correspond to these complete-data models. Model diagnostics: the variance inflation factor (VIF) was < 5 for all models, indicating no significant multicollinearity; residuals passed the Shapiro–Wilk test for normality (P > 0.05); Durbin–Watson values ranged from 1.8 to 2.2, suggesting independent residuals. For univariate correlation analyses (21 tests in total), P-values were corrected for FDR using the Benjamini–Hochberg method (see Section 2.4 and the legend to **Figure 5B**). Multivariable regression was a prespecified secondary adjusted analysis, and thus only raw P-values are reported without additional adjustment for multiple testing. As each independent row in **Table 1** represents a separate multivariable linear regression model (with a single inflammatory cytokine as the dependent variable, a single BCAA as the primary independent variable, and the same set of covariates fixed for adjustment), the adjusted R² and F values presented in each row are the actual fit statistics for that specific model. B: unstandardized regression coefficient; SE: standard error; β: standardized regression coefficient; CI: confidence interval.

Multiple testing correction: Given that this study involved correlation analyses between three serum BCAA indices and seven clinical indicators, totaling 21 independent tests, the Benjamini–Hochberg method was applied to adjust the P-values from Pearson correlation analyses for false discovery rate (FDR) control, with an FDR Q-value < 0.05 considered statistically significant.

It should be noted that bootstrap resampling or cross-validation was not performed for the ROC analysis, primarily because the limited number of positive events (n = 32) would result in a further reduction in the training set sample size, potentially compromising the stability of model estimation.

## Results

### Basic characteristics analysis of the pilot phase small sample

No statistically significant differences were observed between the control group and the DED group in terms of gender, age, BMI, smoking history, alcohol consumption history, or history of underlying diseases (*χ^2^*1 = 0.048, *P* = 0.827; Z1 = -0.655, *P* = 0.513; Z2 = -0.901, *P* = 0.368; *χ^2^*2 = 0.022, *P* = 0.881; *χ^2^*3 = 0.086, *P* = 0.769; *χ^2^*4 = 0.078, *P* = 0.780; *χ^2^*5 = 0.001, *P* = 0.978). Compared to the control group, the DED group showed significantly lower SIT values and significantly higher FL values, and the differences were statistically significant (*t*1 = 22.362, *t*2 = 19.017, *P* < 0.05). No statistically significant differences were found between the two groups in laboratory parameters, including red blood cell count, neutrophil count, hemoglobin concentration, FBG, or HbA1c levels (t1 = 0.400, P = 0.691; t2 = 1.571, P = 0.123; t3 = 0.074, P = 0.942; t4 = 0.549, P = 0.585; t5 = 0.531, P = 0.598, **Table 2**).

**Table 2:** Comparison of general characteristics, ocular symptoms, and laboratory findings between the control group and DED group in the small sample cohort.

Items		Control group (n=22)	DED group (n=26)	*χ^2^*/Z/*t*	P
Gender (cases)	Male	12(54.55)	15(57.69)	0.048	0.827
	Female	10(45.45)	11(42.31)		
Age [years, M (P_25_, P_75_)]		44.00(42.00,48.00)	46.00(41.80,49.00)	-0.655	0.513
BMI [kg·m^-2^, M (P_25_, P_75_)]		24.08(21.00,26.10)	24.37(22.70,25.90)	-0.901	0.368
Smoking history		1(4.45)	2(7.69)	0.022	0.881
Alcohol consumption history		3(13.64)	4(15.38)	0.086	0.769
History of underlying diseases	Hypertension	4(18.18)	5(19.23)	0.078	0.780
	Diabetes	6(27.27)	7(26.92)	0.001	0.978
Ocular surface sign parameters					
SIT (mm/5min)		14.06 ± 1.75	5.23 ± 0.92	22.362	<0.001
FL (scores)		1.61 ± 0.38	6.88 ± 1.25	19.017	<0.001
Laboratory tests					
Red blood cell count (× 10^12^/L)		3.53 ± 0.91	3.43 ± 0.82	0.400	0.691
Neutrophil count (×10^9^/L)		6.12 ± 1.88	6.90 ± 1.56	1.571	0.123
Hemoglobin concentration (g·L^-1^)		139.75 ± 26.15	140.29 ± 24.67	0.074	0.942
FBG (mmol·L^-1^)		4.71 ± 1.42	4.93 ± 1.35	0.549	0.585
HbAlc (%)		5.87 ± 1.36	5.63 ± 1.71	0.531	0.598

### Analysis of untargeted metabolomics for tears

#### Multivariate statistical analysis

Untargeted metabolomic profiling of tear samples from the control and DED groups was performed, with data preprocessing including missing value imputation, unit variance scaling, and quantile normalization to eliminate batch effects and enhance data reliability. Principal component analysis (PCA) revealed that the control (CON) samples (green) clustered predominantly on the right side of the score plot, indicating high metabolic consistency (**Figure 2**). In contrast, the DED samples (red) showed a marked leftward shift and greater dispersion, reflecting substantial heterogeneity in metabolic alterations. The quality control (QC) samples (blue) were located in the central region, partially overlapping with CON but remaining generally distinct, confirming the stability of the analytical system.

### Analysis of differential metabolites

Differential metabolite screening was performed using a dual-threshold criterion: the fold change in expression (|log2FC| > 1) and the FDR Q-value after Benjamini–Hochberg correction (Q < 0.05) were jointly applied as the screening criteria. The P-values presented in the volcano plot are all corrected Q-values [expressed as −log10(Q)], with the cutoff line corresponding to Q = 0.05. Differential metabolites were identified using volcano plots with threshold criteria. As shown in **Figure 3A**, eight metabolites—including N-acetyl ILE, N-acetyl valine, L-arginine, Xanthine, L-ILE, L-pyroglutamate, L-leucine, and L-glycine—were significantly upregulated in the DED group (red markers, log2FC > 1 and FDR-adjusted Q < 0.05), suggesting potential associations with inflammatory responses, oxidative stress, and abnormal tear secretion. In contrast, seven metabolites—L-malate, L-aspartate, citric acid, glutaryl-L-carnitine, aconitic acid, mannose, and L-serine—were significantly downregulated (green markers, log2FC < -1 and Q value < 0.05), indicating impaired tear antioxidant capacity, disturbed energy metabolism, and reduced tear film stability. In addition to the 15 metabolites mentioned above, seven other metabolites showed statistically significant differences between the two groups (P < 0.05) but with |log_2_FC| < 1, indicating relatively small changes in abundance that did not meet the fold-change threshold for selection. Therefore, these metabolites were not included in the subsequent heatmap or pathway enrichment analyses (**Figure 3A**).

**Figure 3:**
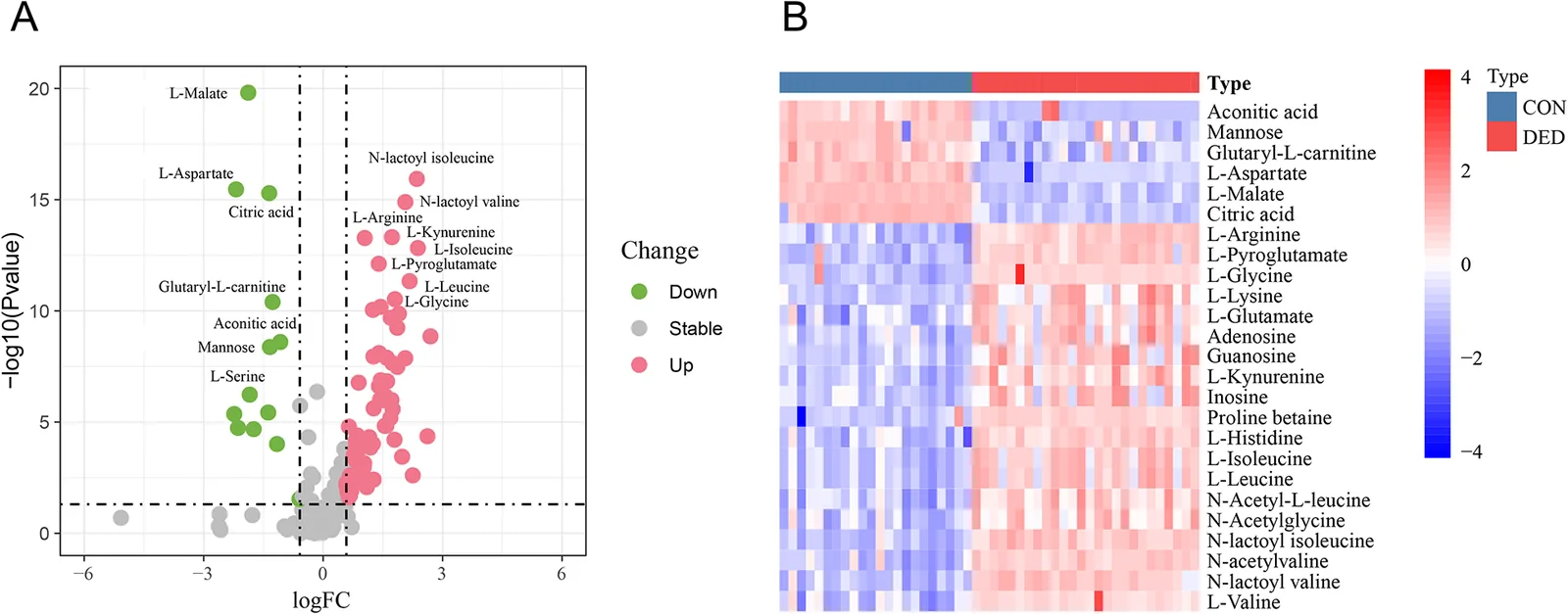
Volcano gram **(A)** and heatmap **(B)** of differential metabolites in tears of the control group and dry eye disease patients (**(A)** Volcano plot of differential metabolites. X-axis: log2-transformed fold change (log2FC), Y-axis: –log10-transformed adjusted Q-value (based on the Benjamini–Hochberg method); Red dots: significantly upregulated metabolites (log2FC > 1 and adjusted Q < 0.05, n = 8); Green dots: significantly downregulated metabolites (log2FC < –1 and adjusted Q < 0.05, n = 7); Gray dots: metabolites that did not meet the differential screening thresholds (n = 133). Dashed lines: screening thresholds (log2FC = ± 1, adjusted Q = 0.05). Among the upregulated metabolites, BCAA family members including L-leucine and L-ILE were identified. **(B)** Clustering heatmap of differential metabolites. Rows: 15 differential metabolites; Columns: 48 individual samples (22 CON + 26 DED), with the color bar at the top of each column indicating group assignment. Colors from blue to red: normalized Z-scores of expression levels from low to high (blue: below the mean by 1 standard deviation; red: above the mean by 1 standard deviation). The heatmap visually demonstrates the discrimination pattern of differential metabolites between the two groups, with L-leucine and L-ILE showing consistently high expression in the DED group. CON, healthy control; DED, dry eye disease; FC, fold change; P-values were adjusted to Q-values using the Benjamini–Hochberg method.).

The heatmap presented in **Figure 3B** further confirmed the group-specific expression patterns of the differential metabolites—notably the elevated levels of L-ILE and L-leucine in the DED group—and highlighted the heterogeneity of metabolic pathway dysregulation in DED patients.

### Pathway enrichment analysis

Pathway enrichment analysis, as shown in **Figure 4**, revealed that the valine, leucine, and ILE biosynthesis pathway was nominally enriched but did not remain statistically significant after FDR correction (raw P = 0.016; FDR Q = 0.058 after Benjamini–Hochberg correction). This suggestive finding from untargeted metabolomics indicated that BCAA metabolic disturbance may represent a metabolic alteration associated with DED observed in the present study, a hypothesis that warrants further validation by targeted LC-MS/MS quantification.

**Figure 4:**
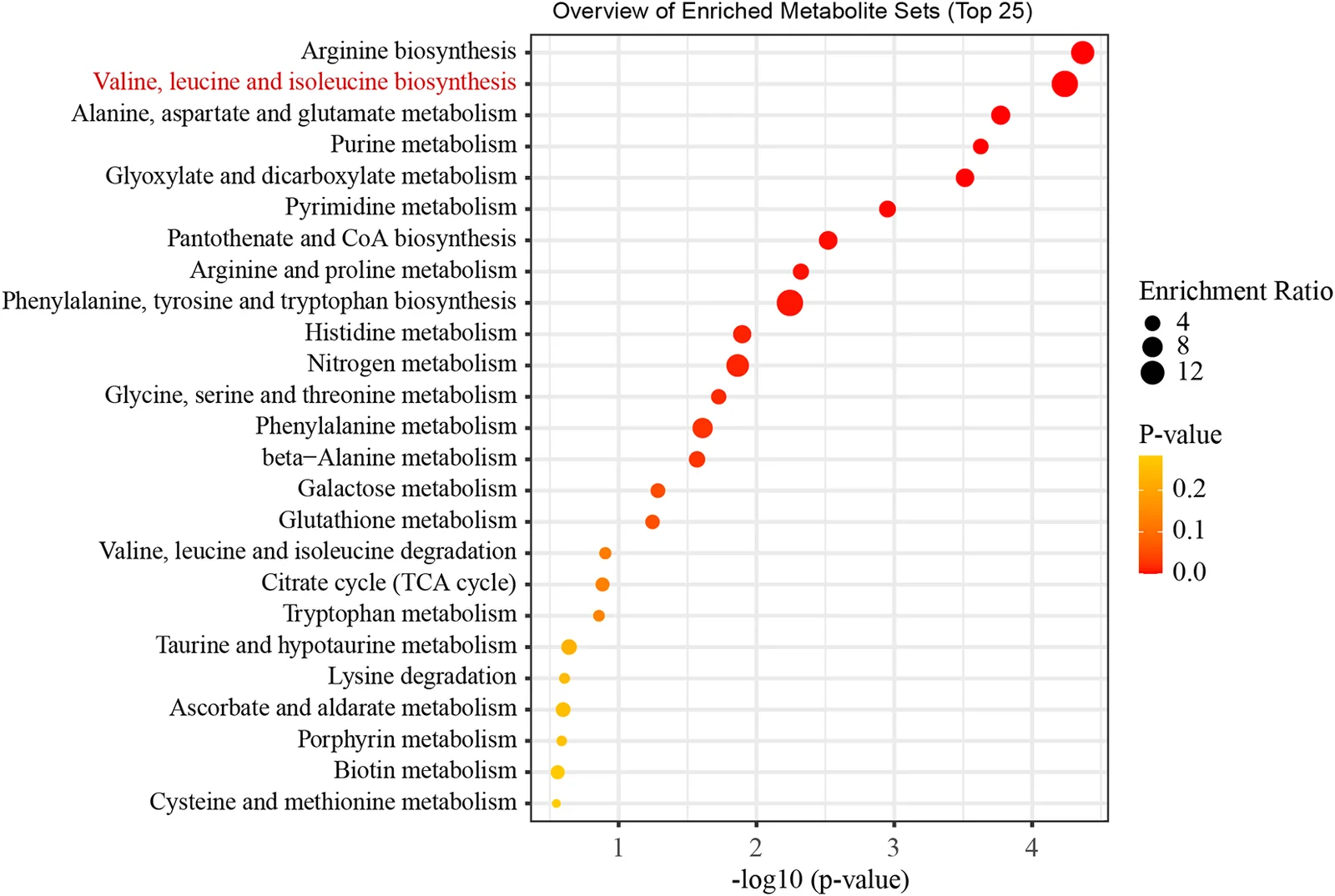
Bubble chart of metabolic pathway enrichment analysis for differential tear metabolites (KEGG pathway enrichment analysis based on the 15 differential metabolites. X-axis: Enrichment Factor (the ratio of the proportion of differential metabolites annotated to a given pathway to the proportion of that pathway annotated in the background database), reflecting the degree of enrichment; Y-axis: KEGG pathway names. Bubble color: –log_10_(P-value) (P based on hypergeometric test), with darker colors (red/orange) indicating higher significance of enrichment; Bubble size: the number of differential metabolites in each pathway, with larger bubbles representing more enriched metabolites. Dashed line: significance threshold of P = 0.05. The valine, leucine and ILE biosynthesis pathway showed a trend toward enrichment (raw P = 0.016; FDR Q = 0.058), suggesting that BCAA metabolic pathways may be involved in tear metabolic reprogramming in DED. Dashed lines represent the significance threshold of P = 0.05 (note: raw P-values are presented here to reflect the original significance of the enrichment trends, whereas the final judgment was based on FDR Q < 0.05).

### Basic characteristics analysis of the large sample in the serum-based sequential exploratory phase

Exploratory analysis of the large independent cohort revealed no statistically significant differences between the control group and DED group in baseline characteristics, including gender, age, BMI, smoking history, alcohol consumption history, or history of underlying diseases (*χ^2^*1 = 0.004, *P* = 0.948; Z1 =-1.838, *P* = 0.066; Z2 =-1.501, *P* = 0.133; *χ^2^*2 = 0.091, *P* = 0.763; *χ^2^*3 = 0.027, *P* = 0.869; *χ^2^*4 = 0.001, *P* = 0.980; *χ^2^*5 = 0.035, *P* = 0.851). Compared to the control group, the DED group showed significantly lower SIT values and significantly higher FL scores, with statistically significant differences (*t*1 = 24.263, *t*2 = 25.533, *P* < 0.05). No statistically significant differences were found between the control group and the DED group in laboratory parameters, including red blood cell count, neutrophil count, hemoglobin concentration, FBG, or HbAlc levels (*t*3 = 0.538, *P* = 0.592; *t*4 = 1.798, *P* = 0.075; *t5* = 1.016, *P* = 0.311; t6 = 0.963, *P* = 0.338, *t*7 = 0.749, *P* = 0.455). In tear inflammatory factors, the DED group showed significantly elevated levels of IL-1β, IL-6, IL-8, hs-CRP, and TNF-α compared to the control group, with statistically significant differences (*t*8 = 63.608, *t*9 = 37.135, *t*10 = 29.201, *t*11 = 38.909, and *t*12 = 79.474, *P* < 0.05, **Table 3**).

**Table 3:** Comparison of general characteristics, ocular symptoms, and laboratory findings between the control group and DED group in the large sample cohort.

Projects		Control group (n=42)	DED group (n=85)	*χ^2^*/Z/*t*	P
Gender (cases)	Male	22(52.38)	44(51.76)	0.004	0.948
	Female	20(47.62)	41(48.24)		
Age [years, M (P_25_, P_75_)]		43.00(42.00,45.00)	42.00(41.00,43.50)	-1.838	0.066
BMI [kg·m-^2^, M (P_25_, P_75_)]		23.06(21.40,25.10)	23.98(22.40,25.40)	-1.501	0.133
Smoking history		4(9.52)	8(9.41)	0.091	0.763
Alcohol consumption history		5(11.90)	11(12.94)	0.027	0.869
History of underlying diseases	Hypertension	6(14.29)	12(14.12)	0.001	0.980
	Diabetes	9(21.43)	17(20.00)	0.035	0.851
Ocular surface sign parameters					
SIT (mm/5min)		14.47 ± 2.83	5.77 ± 1.09	24.263	<0.001
FL (scores)		1.58 ± 0.46	7.02 ± 1.34	25.533	<0.001
Laboratory tests					
Red blood cell count (×10^12^/L)		3.41 ± 0.84	3.50 ± 0.91	0.538	0.592
Neutrophil count (×10^9^/L)		6.32 ± 1.95	6.97 ± 1.90	1.798	0.075
Hemoglobin concentration (g·L^-1^)		146.31 ± 25.04	141.29 ± 26.73	1.016	0.311
FBG (mmol·L^-1^)		4.62 ± 1.32	4.85 ± 1.24	0.963	0.338
HbAlc (%)		6.14 ± 1.51	6.36 ± 1.67	0.749	0.455
IL-1β (ng·L^-1^)		15.16 ± 0.82	69.25 ± 5.47	63.608	<0.001
IL-6 (ng·L^-1^)		10.82 ± 1.93	35.71 ± 4.12	37.135	<0.001
IL-8 (ng·L^-1^)		17.35 ± 2.80	56.07 ± 8.35	29.201	<0.001
hs-CRP (mg·L^-1^)		1.42 ± 0.31	6.29 ± 0.78	38.909	<0.001
TNF-α (ng·L^-1^)		73.92 ± 5.68	263.51 ± 14.91	79.474	<0.001

It should be noted that the sample size of the control group (n = 42) in the serum association exploratory phase did not meet the prespecified minimum requirement of 44 participants per group, which may have somewhat compromised the statistical power for group comparisons of certain indicators with effect sizes approaching the threshold (e.g., baseline variables such as FBG and HbA1c). Nevertheless, for the primary outcome measures in which significant differences were already detected (BCAAs and inflammatory cytokines), the effect sizes were relatively large, and thus the robustness of the conclusions was less likely to be substantially affected by the sample size shortfall.

### Relationship between serum BCAAs and DED severity, ocular symptom indicators, and tear inflammatory factors

BCAAs contents were assessed in serum samples from an independent cohort. The patients in the DED group were divided into a mild to moderate group (64 cases) and a severe group (21 cases) according to the severity of disease. Serum BCAA levels were compared among these two groups and the control group. Both the mild to moderate and severe groups exhibited significantly higher levels of leucine (Leu), valine (Val), and isoleucine (ILE) compared to the control group (*t*1 = 42.286, *t*2 = 32.064, *t*3 = 14.230, *t*4 = 11.291, *t*5 = 42.034, *t*6 = 39.921, all P <0.05). However, no statistically significant differences were observed in serum BCAA levels between the mild to moderate group and severe group (*t*7 = 1.245, *P* = 0.217; *t*8 = 0.395, *P* = 0.694; *t*9 = 1.480, *P* = 0.143, **Figure 5**). These findings suggest that BCAA levels are not significantly correlated with the severity of DED.

**Figure 5:**
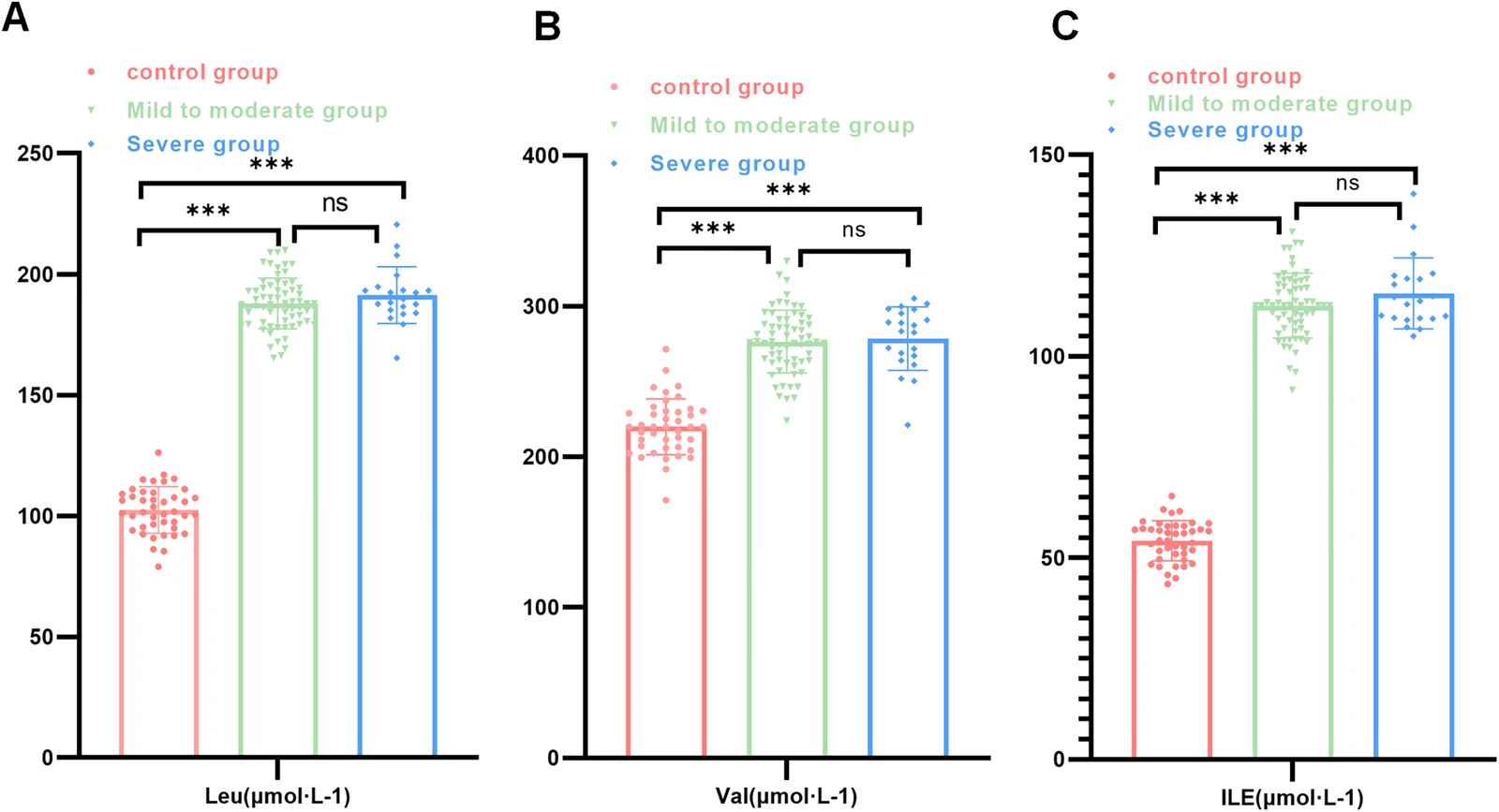
Comparison of serum branched-chain amino acid levels among the three groups (Box plots of serum BCAA levels among the three groups (n = 127, CON = 42, mild-to-moderate DED = 64, severe DED = 21). X-axis: CON, mild-to-moderate DED, and severe DED groups; Y-axis: serum concentration (μmol·L⁻¹), showing Leu, Val, and ILE, respectively. Boxes represent the IQR, with the central line indicating the median, whiskers extending to 1.5 × IQR, and dots representing individual values. Group comparisons were performed using one-way ANOVA with Tukey’s *post hoc* test. ***P < 0.001; ns, P > 0.05. The results showed that serum BCAA levels in the DED groups were significantly higher than those in the CON group, whereas no statistically significant differences were observed between the mild-to-moderate and severe DED groups. **(A)** comparison of Leu in the control group with the mild to moderate group and severe group; **(B)** comparison of Val among three groups; **(C)** comparison of ILE among three groups; ***denoted P < 0.001, *ns* meant no statistical significance).

Pearson correlation analysis revealed that serum BCAAs (Leu, Val, and Ile) were significantly correlated with IL-1β, hs-CRP, and TNF-α in DED patients, but not with IL-6, IL-8, or ocular surface parameters (SIT, FL) (**Figure 6**). Specifically, positive correlations were observed between Leu, Val, and Ile and IL-1β, hs-CRP, and TNF-α (r = 0.57, 0.35, 0.44, 0.54, 0.58, 0.63, 0.66, 0.36, and 0.60, respectively), indicating that serum BCAAs are closely associated with specific tear inflammatory cytokines in DED. All P-values from the 21 correlation analyses were adjusted using the Benjamini–Hochberg method for FDR correction, and the FDR Q-values for all the aforementioned significant correlations were < 0.05. Multivariable linear regression analysis (**Table 1**) revealed that, after adjusting for age, sex, BMI, history of diabetes, and history of hypertension, serum Leu remained independently and positively correlated with IL-1β (β = 0.411, P < 0.001) and hs-CRP (β = 0.387, P < 0.001), whereas its association with TNF-α was not statistically significant (P = 0.823). Val remained independently and positively correlated with IL-1β (β = 0.416, P < 0.001) and hs-CRP (β = 0.335, P = 0.005), but its association with TNF-α was not statistically significant (P = 0.837). Ile remained independently and positively correlated with IL-1β (β = 0.389, P < 0.001) and hs-CRP (β = 0.399, P < 0.001), while its association with TNF-α was not statistically significant (P = 0.785). Collectively, these findings indicate that the associations between serum BCAAs (Leu, Val, and Ile) and IL-1β as well as hs-CRP were independent of common metabolic confounders, with adjusted R² values exceeding 0.46 for the corresponding regression models, suggesting a moderate explanatory power for the variability of these two inflammatory factors. In contrast, the associations with TNF-α were no longer significant after adjusting for confounding factors (adjusted R² approaching zero), suggesting that this inflammatory marker may be influenced by other unmeasured variables, or that its changes in DED are primarily driven by non-BCAA metabolic pathways. All regression models were subjected to collinearity diagnostics (VIF < 5), residual normality tests (Shapiro–Wilk, P > 0.05), and residual independence tests (Durbin–Watson values ranging from 1.8 to 2.2), and all model fitting assumptions were satisfied. Among these, the six models with IL-1β and hs-CRP as dependent variables yielded adjusted R^2^ values ranging from 0.462 to 0.550, with corresponding overall F statistics ranging from 13.022 to 18.111, indicating acceptable goodness-of-fit. In contrast, the three models with TNF-α as the dependent variable showed adjusted R^2^ values close to zero (0.006–0.009), with overall F statistics of 1.085–1.127, suggesting that BCAAs had no explanatory power for TNF-α after controlling for confounding factors. However, it should be noted that, owing to the constraints of retrospective data collection, information on several variables—including dietary structure, renal function, medication exposure, topical ophthalmic treatments, contact lens use history, history of autoimmune diseases, and behavioral factors such as screen time—was not available in this study. As these variables were not included in the multivariable adjustment, residual confounding cannot be ruled out.

**Figure 6:**
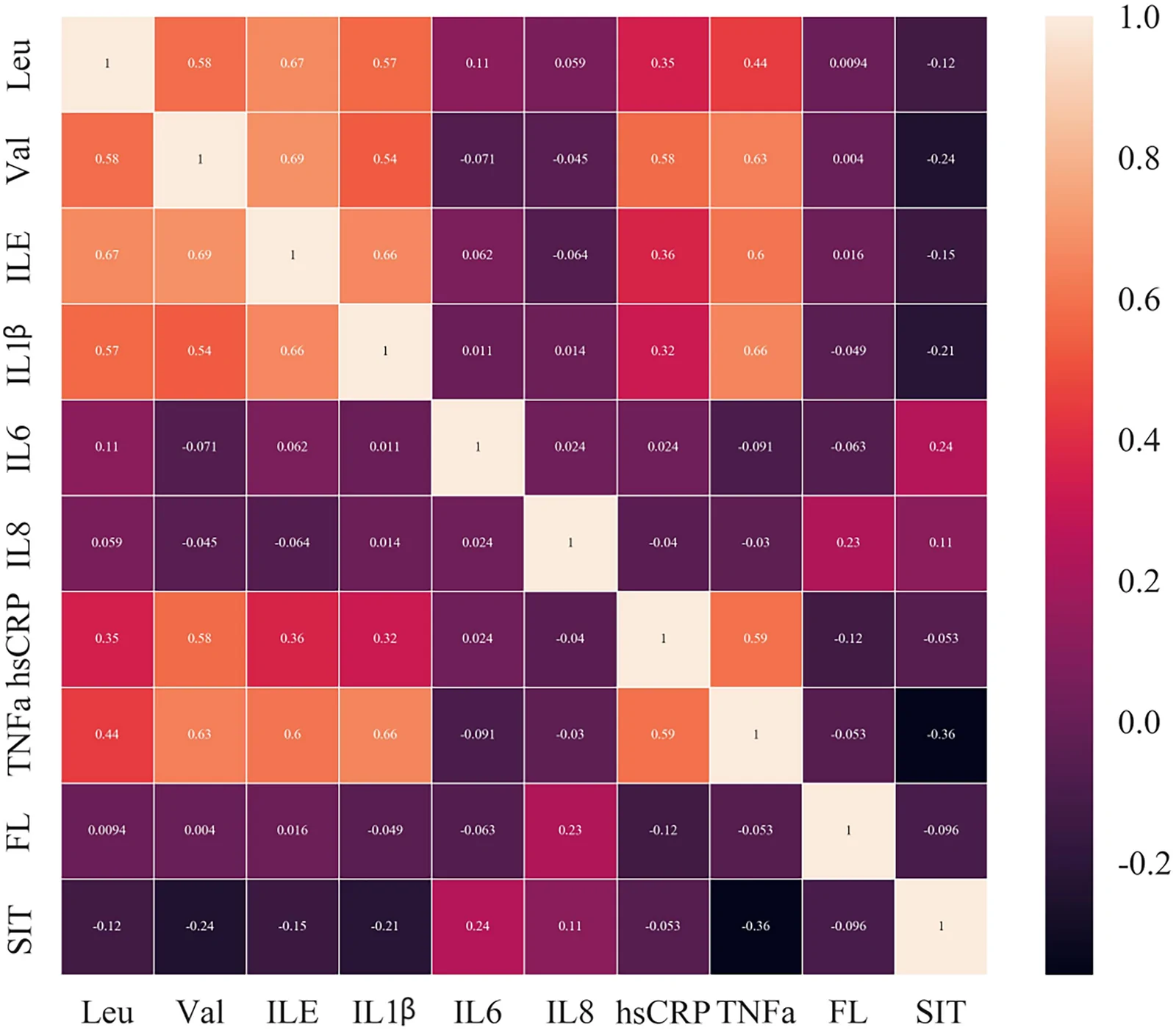
Correlation of serum branched chain amino acids (Leu, Val, ILE) with indicators of ocular surface sign parameters (SIT, FL) and tear inflammatory factors (IL-1β, IL-6, IL-8, hs-CRP, TNF-α) in DED patients (Pearson correlation scatter plot matrix of serum BCAAs and clinical indicators in DED patients (n = 85). Rows (X-axis): serum Leu, Val, and ILE concentrations; Columns (Y-axis): clinical indicators (IL-1β, IL-6, IL-8, hs-CRP, TNF-α, SIT, and FL). Each dot represents an individual DED patient, with blue solid lines representing linear regression fits and gray shaded areas indicating the 95% confidence intervals. Pearson correlation coefficients (r) are shown in the upper-left corner of each subplot, and all P-values were corrected for FDR. The results showed that BCAAs were significantly positively correlated with IL-1β, hs-CRP, and TNF-α (r = 0.35–0.66, all P < 0.05), whereas no significant correlations were observed with SIT or FL (P > 0.05).).

### Relationship between serum BCAAs levels and vision-related quality of life in DED patients

#### VR-QOL evaluation in DED patients

All 85 patients in the DED group were assessed using the National Eye Institute Visual Function Questionnaire (NEI-VFQ-25) to evaluate vision-related quality of life (VR-QOL). The NEI-VFQ-25 comprises 25 items covering 11 dimensions, including general health, ocular pain, near activities, distance activities, social functioning, mental health, role difficulties, dependency, driving, color vision, and peripheral vision. Scores for each dimension were linearly transformed to a scale ranging from 0 to 100, with higher scores indicating better VR-QOL. Based on the application criteria of the NEI-VFQ-25 from previous studies, a score of < 50 in any core dimension (excluding general health and ocular pain) was defined as “poor VR-QOL.” Accordingly, 32 patients were classified into the poor VR-QOL group and the remaining 53 into the good VR-QOL group. This threshold of 50 has been widely used in prior studies as a cutoff to distinguish clinically significant visual impairment from relatively preserved visual function (21). It should be noted that the definition of “poor VR-QOL” as a score of < 50 in any core dimension of the NEI-VFQ-25 was predetermined based on the reference value of this threshold in distinguishing significantly impaired from relatively preserved visual function in the literature. However, this dichotomous classification simplifies functional impairment across different dimensions into a single category, which may mask inter-dimensional heterogeneity. For example, patients with severe impairment in near activities but intact mental health were grouped together with those with impairments across multiple dimensions. This definition is exploratory and may therefore influence the clinical interpretability of the discriminatory performance evaluated by the ROC analysis.

### Comparison of serum BCAAs levels between the poor VR-QOL and the good VR-QOL groups

Compared to the good VR-QOL group, the poor VR-QOL group exhibited significantly higher serum levels of Leu, Val, and ILE, with statistically significant differences (*t*1 = 9.161, *t*2 = 6.222, *t*3 = 7.695; all P < 0.05, **Table 4**).

**Table 4:** Comparison of serum BCAAs between patients with good VR-QOL and poor VR-QOL.

Indicators	Good VR-QOL group (n=53)	Poor VR-QOL group (n=32)	t	P
Leu (μmol·L^-1^)	154.39 ± 14.82	186.37 ± 16.81	9.161	<0.001
Val (μmol·L^-1^)	249.85 ± 17.60	275.92 ± 20.45	6.222	<0.001
ILE (μmol·L^-1^)	101.23 ± 6.79	114.53 ± 9.07	7.695	<0.001

### Multivariable logistic regression analysis of factors associated with poor VR-QOL

Logistic regression analysis was performed by incorporating the differential indicators as variables into the regression model for quantitative assessment and statistical testing. Poor VR-QOL was coded as 1 and good VR-QOL as 0. After variable selection via stepwise regression and validation of model fit, higher serum Leu and Ile levels were significantly associated with increased odds of poor VR-QOL (**Table 5**).

**Table 5:** Multivariable logistic regression analysis of factors associated with poor VR-QOL.

Variable	Assignment	β	SE	Z-value	P-value	OR (95% CI)
Leu	Measured value	0.154	0.048	3.193	0.001	1.166(1.061~1.282)
Val	Measured value	0.043	0.031	1.389	0.165	1.044(0.982~1.110)
ILE	Measured value	0.292	0.098	2.968	0.003	1.339(1.104~1.624)
Age	Measured value	0.018	0.023	0.783	0.433	1.018(0.973~1.065)
BMI	Measured value	0.051	0.082	0.622	0.534	1.052(0.896~1.235)
SIT	Measured value	-0.027	0.085	-0.318	0.751	0.973(0.824~1.147)
FL	Measured value	0.064	0.106	0.604	0.546	1.066(0.866~1.313)
Red blood cell count	Measured value	-0.052	0.174	-0.299	0.765	0.949(0.675~1.334)
Neutrophil count	Measured value	0.038	0.095	0.400	0.689	1.039(0.863~1.250)
Hemoglobin concentration	Measured value	-0.009	0.011	-0.818	0.413	0.991(0.969~1.014)
FBG	Measured value	0.047	0.086	0.547	0.584	1.048(0.884~1.242)
HbAlc	Measured value	0.035	0.068	0.515	0.607	1.036(0.904~1.186)
IL-1β	Measured value	0.014	0.021	0.667	0.505	1.014(0.973~1.057)
IL-6	Measured value	0.008	0.017	0.471	0.638	1.008(0.975~1.042)
IL-8	Measured value	0.006	0.012	0.500	0.617	1.006(0.982~1.031)
hs-CRP	Measured value	0.029	0.041	0.707	0.480	1.029(0.949~1.116)
TNF-α	Measured value	0.005	0.008	0.625	0.532	1.005(0.989~1.021)

The dependent variable was poor VR-QOL, coded as 1 for poor VR-QOL and 0 for good VR-QOL. Therefore, OR > 1 indicates increased odds of poor VR-QOL with increasing values of the corresponding predictor. OR, odds ratio; CI, confidence interval.

### Efficacy of serum BCAAs in discriminating VR-QOL underperformance in DED patients

ROC curve analysis revealed that the area under the curve (AUC) for discriminating poor VR-QOL in DED patients was 0.916 (95% CI: 0.841-0.960) for Leu, 0.837 (95% CI: 0.778-0.908) for Val, and 0.886 (95% CI: 0.814-0.946) for ILE. These results indicated that all three serum BCAAs described above exhibit favorable discriminative ability for poor VR-QOL in DED patients (**Figure 7**). Optimal cutoff values for each indicator were determined based on the Youden index. The optimal cutoff value for Leu was 170.5 μmol/L, yielding a sensitivity of 84.4% and a specificity of 86.8%; for Val, the optimal cutoff was 262.3 μmol/L, with a sensitivity of 78.1% and a specificity of 79.2%; and for ILE, the optimal cutoff was 107.8 μmol/L, corresponding to a sensitivity of 81.3% and a specificity of 83.0%. The positive likelihood ratios were 6.39, 3.75, and 4.78, and the negative likelihood ratios were 0.18, 0.28, and 0.23, respectively.

**Figure 7:**
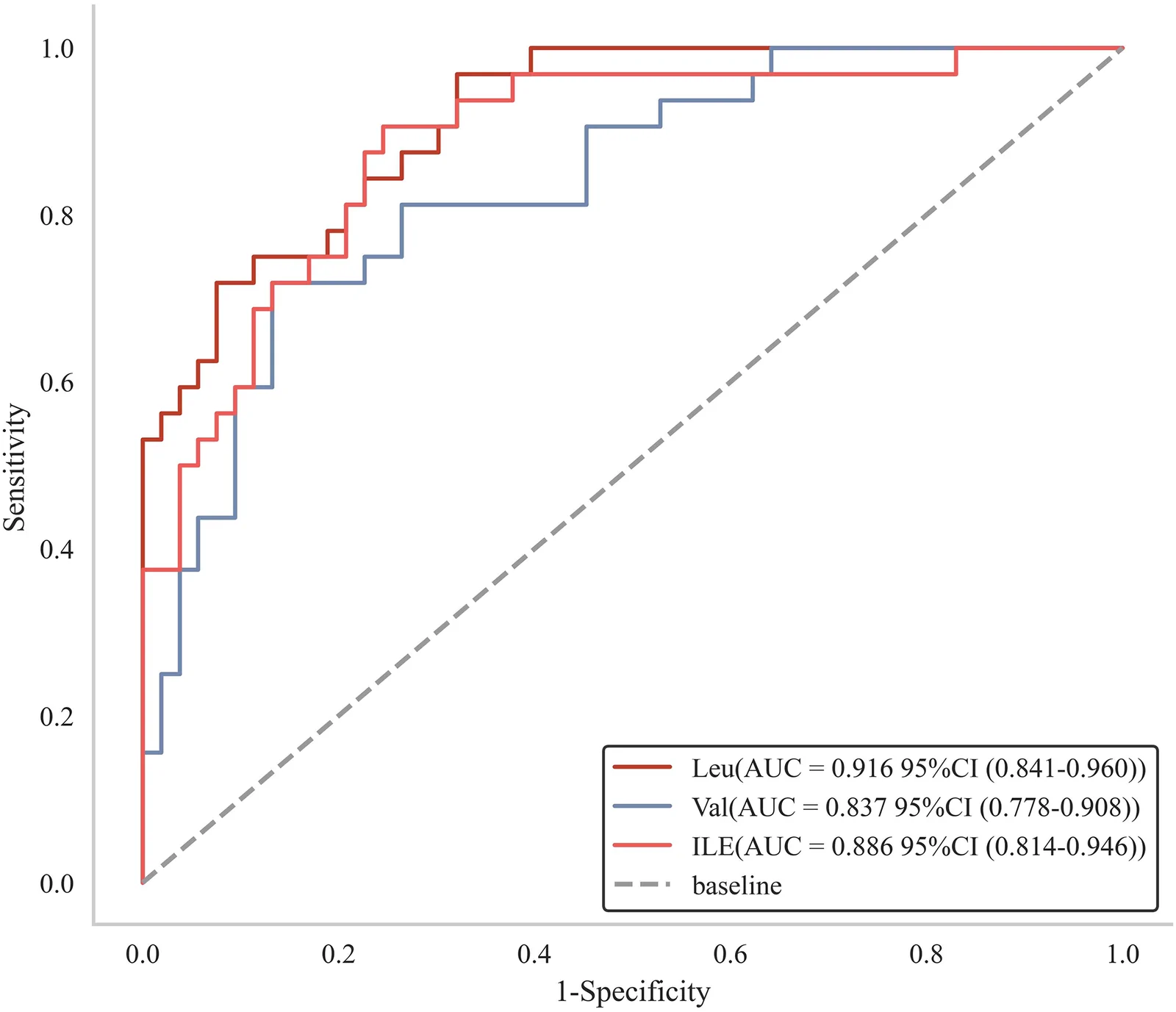
ROC of serum branched-chain amino acids (Leu, Val, ILE) distinguishing poor VR-QOL in DED patients (X-axis: 1 – specificity; Y-axis: sensitivity. Red solid line: Leu (AUC = 0.916, 95% CI: 0.841–0.960); green dashed line: Val (AUC = 0.837, 95% CI: 0.778–0.908); blue dotted line: ILE (AUC = 0.886, 95% CI: 0.814–0.946); gray diagonal line: reference line (AUC = 0.5). The AUC values of BCAAs for distinguishing poor VR-QOL (a score < 50 in any core dimension of the NEI-VFQ-25) were all > 0.83, suggesting favorable apparent discriminative ability. However, these results have not been validated and require independent cohort validation to determine whether overfitting exists.) CON, healthy control; DED, dry eye disease; IQR, interquartile range; IL, interleukin; hs-CRP, high-sensitivity C-reactive protein; TNF-α, tumor necrosis factor-α; SIT, Schirmer I test (mm/5 min); FL, corneal fluorescein staining (scores); VR-QOL, vision-related quality of life. Group comparisons were performed using one-way ANOVA with Tukey’s *post hoc* test; *P < 0.05, **P < 0.01, ***P < 0.001; ns, P > 0.05. Correlation analyses were performed using Pearson’s method, with r and P values indicated in the corresponding subplots; AUC values are presented with 95% confidence intervals.

## Discussion

Recent research on DED pathogenesis has suggested that reduced tear secretion and tear compositional imbalances may contribute to the onset and progression of the disease (22). This study, through tear metabolomics analysis, validated abnormalities in the tear BCAAs pathway (consistent with reports by Mingxia X et al. (23)) and provided new exploratory evidence for the association between serum BCAAs and ocular surface inflammation. Untargeted tear metabolomics analysis in a small sample identified significant differences in metabolic profiles between healthy individuals and DED patients, suggesting that DED may be associated with marked alterations in tear metabolites. Further analysis revealed that in the DED group, 8 metabolites were significantly upregulated and 7 were significantly downregulated. This indicates a systematic imbalance in the tear metabolomic profiles of DED patients, and the abnormalities in the key metabolites and pathways could provide a theoretical basis for understanding disease mechanisms and developing diagnostic biomarkers. This study also highlighted a trend toward enrichment of valine, leucine and ILE biosynthesis pathways in the DED group (raw P = 0.016; FDR Q = 0.058), suggesting that the systemic dysregulation of the BCAA metabolic network may be linked to the development of DED. This finding suggests an exploratory direction that warrants further evaluation: whether BCAA metabolic pathways are involved in the pathophysiology of DED remains to be verified by future mechanistic studies.

In recent years, the role of systemic metabolic disorders in the onset and progression of ocular diseases has attracted increasing attention. The advancement of metabolomic technologies has enabled researchers to systematically delineate the metabolic landscape of the ocular surface microenvironment and to explore the potential bridge between systemic metabolic status and ocular surface health (24). In the field of DED, previous studies using tear metabolomics have revealed abnormalities in lipid, amino acid, and energy metabolic pathways (25). However, most studies have been confined to descriptive comparisons of local tear metabolites and have failed to link local metabolic alterations with systemic metabolic status. In the broader spectrum of ocular diseases, systemic metabolic factors have been implicated in the pathophysiology of various ocular conditions. In diabetic retinopathy, disturbances in branched-chain amino acid metabolism have been associated with retinal neurovascular damage. In age-related macular degeneration, aberrant lipid metabolism has been shown to interact with complement system activation. Moreover, patients with glaucoma exhibit systemic alterations in serum amino acid profiles (26). These lines of evidence suggest that systemic metabolic dysregulation may affect ocular tissues through multiple pathways, including systemic low-grade chronic inflammation, insulin resistance, and oxidative stress. However, studies on the association between systemic BCAA metabolism and ocular surface inflammation in DED remain scarce. Against this background, the present study attempted to explore the metabolic association between systemic BCAA levels and DED from a metabolomic perspective.

Key findings: The principal observations of this study were as follows: (1) Untargeted tear metabolomics revealed a trend toward enrichment of the valine, leucine and isoleucine biosynthesis pathway in the DED group; (2) in the serum-based sequential exploratory analysis using an independent serum cohort, serum levels of Leu, Val, and Ile were significantly elevated in DED patients compared with controls; (3) serum BCAAs showed positive correlations with tear inflammatory cytokines (IL-1β, hs-CRP, and TNF-α) at the population level, whereas no significant associations were observed with ocular surface parameters including SIT and FL; and (4) serum BCAAs were associated with VR-QOL at the population level.

Reasonable interpretation and speculation of the results: The positive correlation between elevated serum branched-chain amino acids (BCAAs) and tear inflammatory cytokines may reflect a potential link between systemic metabolic disturbances and ocular surface inflammation. Previous animal studies have demonstrated that BCAAs can promote the expression of inflammatory cytokines via the mTOR signaling pathway, thereby providing a possible biological framework to account for the correlations observed in our study. However, it should be emphasized that the present study did not directly assess mTOR pathway activity, oxidative stress biomarkers, or immune cell infiltration; therefore, the above mechanistic interpretation remains speculative and is based solely on literature-derived evidence. The negative findings, namely the lack of significant correlations between serum BCAAs and either SIT or FL, suggest that BCAAs primarily reflect systemic metabolic status rather than the severity of local ocular surface pathological damage. Consequently, serum BCAAs are not suitable as biomarkers for evaluating DED severity, and their potential utility may be limited to early risk identification in high-risk populations. Furthermore, alternative explanations for the elevated serum BCAAs observed in this study should be considered, independent of the pathological processes underlying DED. Serum BCAA levels are influenced by a variety of metabolic and lifestyle factors, including dietary protein intake, insulin resistance, metabolic syndrome, systemic low-grade inflammation, and renal dysfunction. Given the retrospective design of this study, data pertaining to the aforementioned variables were not collected; consequently, the observed association between serum BCAAs and DED cannot exclude the possibility that it is co-driven by these unmeasured confounders. It is more plausible that elevated BCAAs represent an epiphenomenon of systemic metabolic dysregulation rather than a specific driver of ocular surface inflammation in DED.

Previous studies have found that elevated serum BCAAs levels are involved in systemic inflammatory responses, and animal experiments have suggested that BCAAs may promote the expression of inflammatory cytokines via the mTOR signaling pathway (27–30). Ocular surface inflammation is an important pathological basis of DED (31, 32). At the population level, the present study observed positive correlations between serum BCAAs and tear levels of IL-1β, hs-CRP, and TNF-α, thereby providing indirect clues regarding the relevance of the aforementioned mechanisms in DED. However, it must be clearly emphasized that the proposed mechanisms—including mTOR pathway activation, oxidative stress, and immune cell infiltration—were not directly experimentally validated in the present study. The above hypotheses remain speculative, based solely on previous literature, and await confirmation or refutation by future mechanistic investigations.

The primary pathological basis of DED is the disruption of tear film stability. SIT and FL are the main clinical indicators for assessing tear film stability and can reflect the severity of ocular discomfort in DED patients. Lower SIT values and higher FL scores indicate worse tear film stability and more severe clinical signs (33). Of particular note, in this study, serum BCAA levels did not differ significantly between patients with mild-to-moderate and severe DED, nor were they significantly correlated with local ocular surface parameters such as SIT and FL. This negative finding is not unexpected—it directly indicates that BCAAs reflect systemic metabolic disturbances rather than the severity of local ocular surface pathological damage, and therefore cannot serve as biomarkers for DED disease severity. This apparent contradiction carries decisive implications for the clinical positioning of BCAAs. Elevated serum BCAA levels may suggest an individual’s susceptibility to DED or reflect systemic metabolic states that frequently coexist with DED—such as insulin resistance and chronic low-grade inflammation. However, once the disease is diagnosed, its local severity appears to be more dependent on factors specific to the ocular surface, including local inflammatory cascades, neural damage, and visual habits, rather than on systemic BCAA metabolic status. Consequently, the potential clinical application of serum BCAAs should be strictly confined to early risk screening in high-risk DED populations, rather than serving as a tool for assessing or staging disease severity. Reclassifying BCAAs as “metabolism-related indicators of disease susceptibility” rather than “markers of disease severity” represents a reasonable and necessary response to the negative findings of this study, and also points toward future research directions: the value of BCAAs in DED lies in early identification, not in disease monitoring. Furthermore, it should be emphasized that, given the cross-sectional design of this study, causal relationships between BCAAs and DED cannot be established. The precise role of BCAAs in the pathogenesis of DED—whether as a causative factor, an epiphenomenon, or a compensatory alteration—remains to be elucidated through prospective cohort studies and basic mechanistic investigations. It is premature to define serum BCAAs as potential biomarkers; a more accurate description would be “blood parameters metabolically associated with DED.” Currently, the pathogenesis of DED remains incompletely understood. It is a chronic ocular surface disorder with multifactorial origins, often characterized by significant ocular discomfort. Progressive worsening of the condition may lead to visual impairment and reduced visual acuity, severely impacting patients’ VR-QOL. The ROC analysis in this study showed that serum BCAA levels exhibited a high apparent discriminative ability for poor VR-QOL in DED patients (AUC: 0.837-0.916). However, it should be noted that this analysis was based on a single-center cohort (n = 85), and neither internal cross-validation nor external independent validation was performed. The relatively high AUC values may therefore be influenced by sample-specific characteristics and the risk of overfitting, and the robustness of its screening utility awaits confirmation in independent cohorts. Accordingly, the present findings should be regarded as exploratory, and their generalizability requires further verification in larger, multicenter cohorts prior to clinical translation. Furthermore, for the purposes of clinical interpretability and ROC analysis, VR-QOL was dichotomized into two categories (poor vs. good) in this study. Although this approach offers intuitive clarity and clinical feasibility, it may entail a loss of informational granularity inherent in the NEI-VFQ-25 score as a continuous variable. Future investigations could employ multiple linear regression or ordinal logistic regression to analyze VR-QOL as a continuous or ordinal outcome, thereby enabling a more comprehensive characterization of the dose–response relationships between BCAAs and various dimensions of vision-related quality of life.

Strengths and positioning of the study: a novel two-stage sequential design. This study first employed untargeted metabolomics of tear fluid to screen for differential metabolites and enriched pathways, followed by targeted quantification of systemic BCAA alterations in an independent serum cohort. This “discovery–association exploration” strategy represents, to our knowledge, the first attempt in the field of metabolomics research in DED. By simultaneously reporting BCAA metabolic abnormalities at both the tear and serum levels in DED, our study—demonstrating a trend toward enrichment of the BCAA biosynthesis pathway in tears and overall upregulation of leucine, valine, and ILE in serum—opens new avenues for investigating the metabolic mechanisms underlying DED. Emphasis on patient-reported outcomes. By incorporating vision-related quality of life (NEI-VFQ-25) as a core outcome measure, this study directly addresses patients’ concerns regarding daily visual function, thereby enhancing its clinical translational value. Strict multi-tiered quality control. In the metabolomic profiling, a multi-tiered quality control system was established, comprising blank controls, internal standard calibration, and quality control (QC) samples. The tight clustering of QC samples ensures the reliability and reproducibility of the data.

Within the framework of biomarker validation, this study resides at the early exploratory phase of biomarker discovery. According to the Pepe three-phase framework and the FDA-NIH BEST (Biomarkers, EndpointS, and other Tools) classification system: at the analytical validation level, the detection methods for tear and serum samples have been established with satisfactory QC performance; at the clinical validation level, robust associations have yet to be confirmed in large-scale, multicenter, prospective cohorts; and at the clinical utility level, the ROC analyses lack cross-validation in independent cohorts, precluding any definitive conclusions regarding screening utility. Therefore, the present findings should be appropriately positioned as “exploratory evidence for a metabolic association between BCAAs and DED,” and future investigations should progressively advance along the sequential logic of biomarker validation.

Innovations, limitations, and future perspectives of this study: The study employed a two-stage independent cohort design, with the pilot phase based on untargeted metabolomics of tear fluid (n = 48) and the serum-based sequential exploratory phase based on targeted serum BCAA quantification (n = 127). Importantly, the analytical subjects in the two stages did not overlap, and paired tear–serum measurements were not performed in the same individuals. Consequently, the relationship between the two stages does not constitute a conventional “discovery–independent validation” paradigm; rather, it represents an exploratory, sequential analysis across different biological matrices. As a result, a quantitative correlation between systemic BCAA levels and local ocular surface metabolic alterations cannot be directly established at the individual level. The sample size of the control group (n = 42) in the serum association exploratory phase did not meet the prespecified minimum requirement of 44 participants per group. Although the DED group (n = 85) was adequately sized, the insufficient number of controls resulted in the statistical power of some tests being lower than originally intended, which may have particularly affected the ability to detect differences of moderate effect sizes. This suggests that some of the negative findings in the present study may be constrained by limited statistical power due to the sample size shortfall and should therefore be interpreted with caution. Future studies are warranted to further validate our findings in larger, independent cohorts with balanced group sizes. As a retrospective, single-center observational study, selection bias is difficult to avoid. The enrolled population consisted predominantly of Han Chinese outpatients with relatively severe disease, which limits the generalizability of our findings. Moreover, constrained by the completeness of medical records, we were unable to obtain information on several potential confounding variables that may independently influence serum BCAA levels or DED severity, including dietary composition, use of branched-chain amino acid supplements, medication exposure (including topical ophthalmic treatments), contact lens wear history, autoimmune disease status, and screen time. Consequently, residual confounding cannot be excluded and may affect the accuracy of our effect estimates. The tear metabolomic profiling was performed using untargeted semi-quantitative analysis, and the identification of BCAA-related differential metabolites relied primarily on MSI Level 2 (database matching), without targeted LC-MS/MS absolute quantification. Consequently, the exact concentrations of BCAAs in tear fluid could not be determined. The ROC analyses were based on a single-center, small-sample cohort with only 32 positive events, and neither internal cross-validation nor external independent validation was performed. The relatively high AUC values (e.g., 0.916 for leucine) are therefore susceptible to overfitting, and the robustness of their apparent discriminative ability requires confirmation in independent large-sample cohorts. Furthermore, the sample sizes in the exploratory phase (22 vs. 26) and in the severe DED subgroup (n = 21) were relatively small, which may limit the statistical power for stratified analyses. Additionally, some negative results following multiple comparison correction may still be attributable to insufficient statistical power. This study employed a cross-sectional observational design, and no cellular or animal experiments were conducted to elucidate the causal mechanisms linking BCAA metabolic abnormalities to ocular surface inflammation. The proposed pathway hypotheses, such as involvement of the mTOR signaling cascade, are derived solely from literature-based inference and await confirmation through future mechanistic investigations. In summary, the present findings should be interpreted as exploratory in nature, and their clinical translational value requires further substantiation through paired-sample prospective cohort studies, multicenter validation, and mechanistic experiments.

## Conclusion

In summary, the pilot-phase untargeted tear metabolomic analysis suggested BCAA-related metabolic alterations in patients with DED. The exploratory analysis in the large-sample independent cohort during the progressive analysis phase revealed that serum levels of Leu, Val, and Ile were positively correlated with specific tear inflammatory cytokines (IL-1β, hs-CRP, and TNF-α) in DED patients. However, no significant correlation was observed between serum BCAAs indicators and either disease severity or ocular surface sign parameters in DED patients. Furthermore, changes in serum BCAAs levels were closely associated with VR-QOL in DED patients. At the population level in our cohort, serum BCAAs (leucine, valine, and ILE) showed significant associations with certain tear inflammatory cytokines (IL-1β, hs-CRP, and TNF-α) and with vision-related quality of life, but not with clinical measures of DED severity, including DEWS II grading, SIT, and FL. These findings suggest a potential metabolic link between systemic BCAA dysregulation and ocular surface inflammation in DED. However, whether BCAA metabolic pathways are actively involved in the pathophysiology of DED remains to be validated through mechanistic studies, and the clinical relevance of these observations warrants further investigation.

## Data Availability

The original contributions presented in the study are included in the article/supplementary material. Further inquiries can be directed to the corresponding author.
